# The Non-Peptidic Part Determines the Internalization Mechanism and Intracellular Trafficking of Peptide Amphiphiles

**DOI:** 10.1371/journal.pone.0054611

**Published:** 2013-01-17

**Authors:** Dimitris Missirlis, Tambet Teesalu, Matthew Black, Matthew Tirrell

**Affiliations:** 1 Department of Bioengineering, University of California, Berkeley, California, United States of America; 2 Department of Biophysical Chemistry, Physical Chemistry Institute, University of Heidelberg, Heidelberg, Germany; 3 Department of New Materials and Biosystems, Max Planck Institute for Intelligent Systems, Stuttgart, Germany; 4 Laboratory of Cancer Biology, Institute of Biomedicine, Centre of Excellence for Translational Medicine, University of Tartu, Tartu, Estonia; 5 Cancer Center, Sanford-Burnham Medical Research Institute, La Jolla, California, United States of America; 6 Institute for Molecular Engineering, University of Chicago, Chicago, Illinois, United States of America; Consejo Superior de Investigaciones Cientificas, Spain

## Abstract

**Background:**

Peptide amphiphiles (PAs) are a class of amphiphilic molecules able to self-assemble into nanomaterials that have shown efficient *in vivo* targeted delivery. Understanding the interactions of PAs with cells and the mechanisms of their internalization and intracellular trafficking is critical in their further development for therapeutic delivery applications.

**Methodology/Principal Findings:**

PAs of a novel, cell- and tissue-penetrating peptide were synthesized possessing two different lipophilic tail architectures and their interactions with prostate cancer cells were studied *in vitro*. Cell uptake of peptides was greatly enhanced post-modification. Internalization occurred *via* lipid-raft mediated endocytosis and was common for the two analogs studied. On the contrary, we identified the non-peptidic part as the determining factor of differences between intracellular trafficking and retention of PAs. PAs composed of di-stearyl lipid tails linked through poly(ethylene glycol) to the peptide exhibited higher exocytosis rates and employed different recycling pathways compared to ones consisting of di-palmitic-coupled peptides. As a result, cell association of the former PAs decreased with time.

**Conclusions/Significance:**

Control over peptide intracellular localization and retention is possible by appropriate modification with synthetic hydrophobic tails. We propose this as a strategy to design improved peptide-based delivery systems.

## Introduction

Targeted delivery of macromolecular or supramolecular structures *in vivo* to a desired tissue, cell population or intracellular compartment constitutes a major challenge towards development of effective therapeutic and/or diagnostic modalities [1]. Peptides can serve as targeting agents for drug delivery systems [1], [2] and additionally mediate intracellular delivery by efficiently crossing membrane barriers. For example, cell-penetrating peptides (CPPs) have a unique ability to induce internalization of drug formulations in a variety of cells *in vitro* [3]. Ideally, tissue-specific targeting and cell-specific internalization would be combined in one sequence. Recently, screening of phage libraries led to the identification of the C-terminal peptide motif R/K-XX-R/K (C-*end rule* or CendR motif) as a critical element in neuropilin-1 (NRP-1) mediated internalization, targeting, and vascular and tissue penetration [4], [5].

The favorable tumor-homing and cell penetration properties of CendR peptides led us to explore means for their integration in nanoscale drug delivery systems via self-assembly. Peptides modified with hydrophobic, lipid-like tails known as ‘peptide amphiphiles’ (PAs) can be used as building blocks for the production of self-assembled nanostructures [6] or as functional coatings on preformed nanostructures [7], [8]. The physicochemical properties of the hydrophobic tails and the interactions between peptide headgroups specify the supramolecular geometry [9]. For example, interposition of poly(ethylene glycol) between tissue-specific targeting peptides and a di-stearyl lipid tail favors formation of small spherical micelles [10]. Such micelles demonstrated peptide-mediated, *in vivo* homing to atherosclerotic plaques and to different tumors in mice [11]–[13]. However, as interactions between the PAs are physical in nature, the structures possess an inherent dynamic character that clearly poses an issue of stability. Indeed, *in vitro* studies have shown that in presence of albumin and lipid membranes micelle disassembly occurs within minutes [14], [15]. As a consequence, PA internalization occurs following micelle disassembly and monomer insertion to the plasma membrane [15], [16].

Here we studied the *in vitro* internalization and trafficking of PAs presenting the prototypic CendR peptide, RPARPAR [4]. Our data indicate that the lipid-anchor and not the peptide is the key determinant factor for internalization and differences in its structure result in altered subcellular trafficking of the amphiphiles. Our results have key design implications for exploiting the potential of PAs in drug delivery applications.

## Results

### Design of Amphiphiles used in this Study

**Figure 1:**
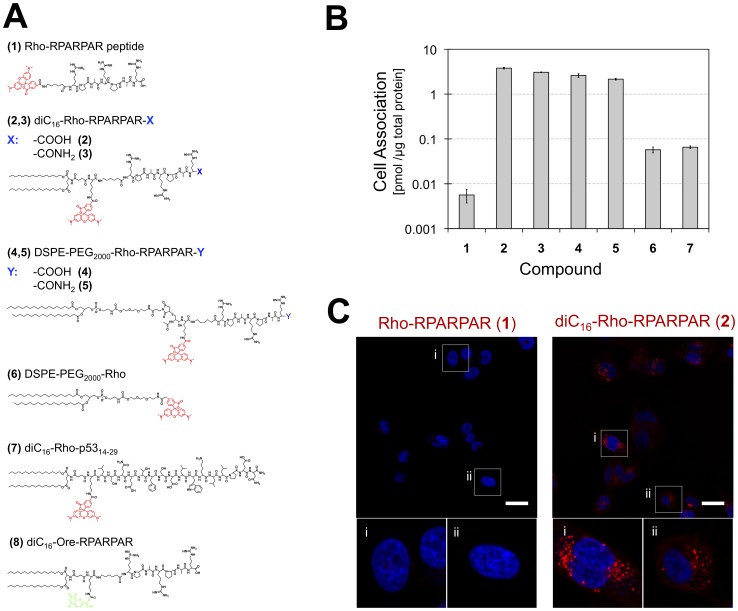
RPARPAR PAs internalize in PPC-1 cells in vitro to a higher extent than the peptide. (A) Chemical structures of fluorescent peptides, peptide amphiphiles and control amphiphiles used in this study. (B) Quantification of cell-associated peptides, PAs and control amphiphile (concentration: 10 µM) after 1-hour incubation with PPC-1 cells revealed higher association for both types of RPARPAR PAs (**2**, **4**) compared to peptide (**1**). Cell association was similar for RPARPAR PAs with carboxylated (**2**, **4**) or amidated C-terminus (**3**, **5**). Control amphiphile **6** and PA **7** showed lower association compared to RPARPAR PAs (**2–5**) Mean values and SEM are presented. (C) Confocal micrographs acquired under the same microscope settings confirmed elevated cellular uptake of PA **2** compared to peptide (**1**) and displayed a punctate intracellular fluorescence pattern. Nuclei stain (blue): Hoechst 33342; Scale bars: 40 µm.

Peptide amphiphiles (PAs) of carboxyl-terminated RPARPAR peptide were synthesized with two different synthetic lipid tails. The di-palmitic tail (diC_16_) [17] was conjugated to the peptide via an amide bond on the resin and the resulting PA was fluorescently labeled with rhodamine (**2**) or oregon514 dye (**8**) (Figure 1A). Alternatively, the commercially available lipid DSPE-PEG_2000_-Maleimide consisting of two stearyl tails linked to poly(ethylene glycol) was attached via a maleimide-thiol bond to a cysteine-containing RPARPAR peptide in solution, which was then labeled with rhodamine (**4**) (Figure 1A). Control amphiphiles included: a) amide-terminated RPARPAR PAs of both types (**3**: diC_16_, **5**: DSPE-PEG_2000_), b) a PA composed of a non-CendR, 16-mer, membrane-impermeable peptide (p53_14–29_) modified with the diC_16_ tail (**7**) [15], and c) a rhodamine-labeled DSPE-PEG_2000_ amphiphile (**6**) (Figure 1A).

### RPARPAR Modification with Hydrophobic Tail Greatly Enhances Association with PPC-1 Cells

PAs **2** and **4** exhibited more than 3 orders of magnitude higher association with PPC-1 cells *in vitro*, compared to peptide **1** (Figure 1B). RPARPAR phage particles and quantum dots bind to cell surface NRP-1 and internalize in PPC-1 cells only if the C-terminus of the peptide is carboxylated [4]. In contrast, amidated RPARPAR PAs **3** and **5** exhibited comparable cell association to carboxylated ones (Figure 1B). These results indicated that the hydrophobic tail of PAs promotes their cell association, which was not primarily dependent on NRP-1 binding. Indeed, association of PA **2** with M21 cells (which do not express NRP-1) was in the same order of magnitude as that calculated for PPC-1 cells (7.1±0.4 pmol/µg total protein; mean±SEM) and orders of magnitude higher than that of **1** (0.002±0.001 pmol/µg total protein; mean±SEM). Control amphiphiles without a peptide (**6**) or PAs with the cell-impermeable peptide p53_14–29_ (**7**) [15] associated with cells at lower levels than RPARPAR PAs suggesting a dependence of PA headgroup in cell association (Figure 1B).

### PAs Localize in Intracellular Vesicles

Confocal microscopy confirmed the elevated cell-association of PA **2** compared to peptide **1** (Figure 1C). After 1-hour incubation, the majority of PA **2** exhibited an intracellular punctate fluorescence pattern indicating vesicular localization (Figure 1C). At early time points (1 & 10 min) PA **2** localized primarily on the plasma membrane (Figure S1) indicating that internalization occurs following PA association with the membrane. Qualitatively, there were no differences in intracellular fluorescence distribution between: *i)* PA **4** and the control amphiphile lacking the peptide sequence (**6**), *ii)* carboxylated (**2**) and amidated (**3**) RPARPAR PAs and *iii)* PAs **2** and **8**, carrying a different fluorescent label (Figure S2). Initial imaging experiments were performed using epiflurescent microscopy on live cells to exclude fixation artifacts (Figure S3). Collectively, these data show that following initial plasma membrane association a large fraction of PAs internalize into intracellular vesicles independent of peptide presence or the nature of fluorescent label.

### PA Internalization Occurs Primarily through Clathrin-independent Carriers

**Figure 2:**
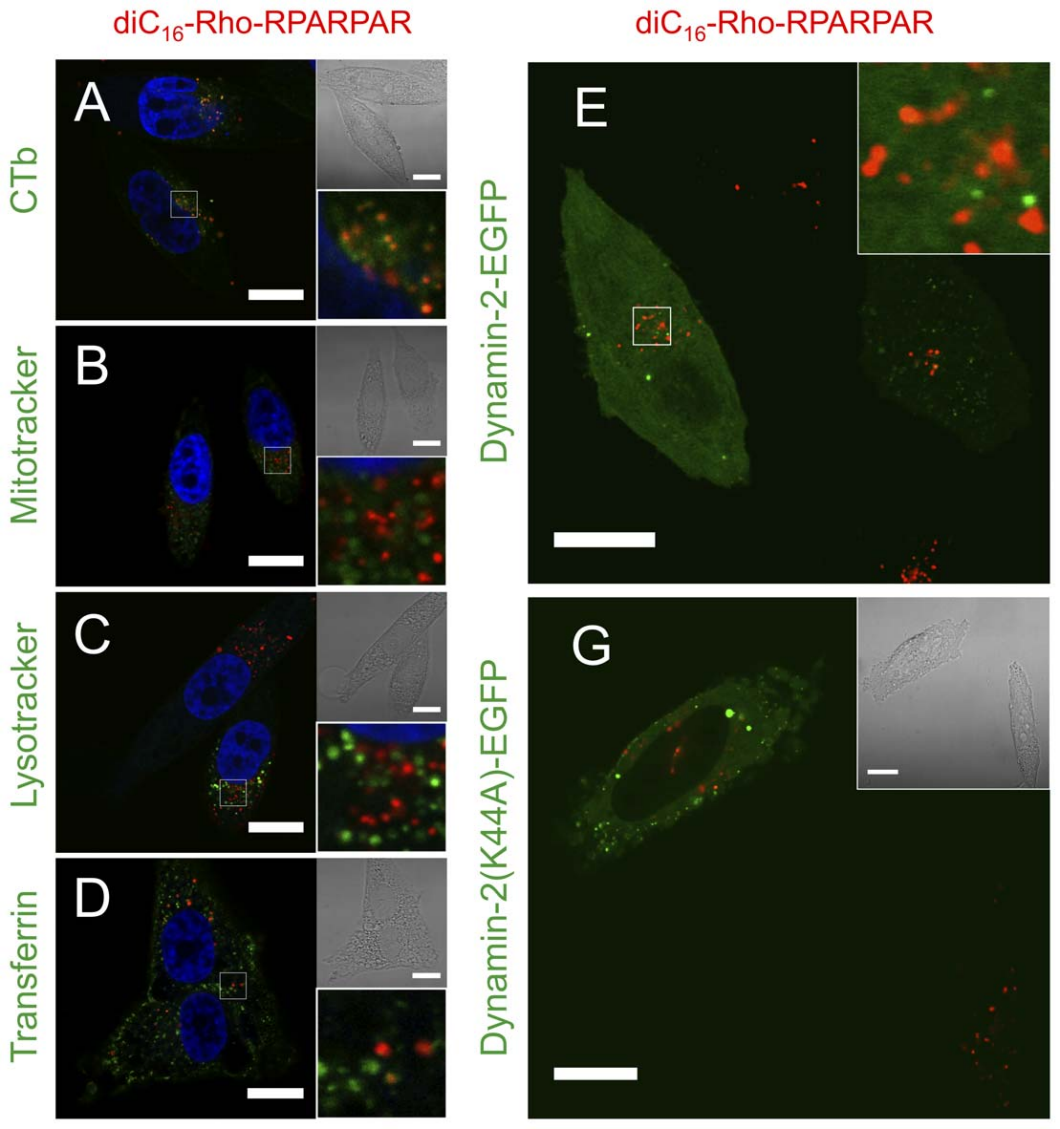
diC16-Rho-RPARPAR (2) co-localizes with CTb following 1-hour incubation in PPC-1 cells and is internalized in a dynamin-2-independent manner. (A–D) PPC-1 cells incubated with PA **2** (10 µM) co-localized with CTb (A; yellow indicates co-localization) but not with mitochondria (B; Mitotracker) or lysosomes (C; Lysotracker). A small fraction of intracellular vesicles were positive for both PA **2** and transferrin (D). PPC-1 cells were transfected with EGFP-coupled dynamin-2 (E) or a dominant negative dynamin-2 mutant (G). 24 hours after transfection, cells were incubated for 1 hour with 10 µM PA **2**. Absence of co-localization with dynamin-2 (E) and internalization in PPC-1 cells expressing the dominant negative dynamin-2 mutant (G) indicate that PA **2** enters cells in a dynamin-2-independent manner. Nuclei stain (blue): Hoechst 33342; Scale bars: 20 µm.

To identify the subcellular compartment to which RPARPAR PAs are directed, PPC-1 cells were co-incubated with PAs and different internalization pathway markers (Figure 2A–D). Co-localization of PA **2** with cholera toxin subunit B (CTb) after 1-hour incubation revealed substantial overlap, whereas there was only a limited overlap with transferrin and no overlap with lysotracker or mitotracker (Figure 2A–D). Co-localization of PAs with CTb was evident as early as 10 minutes after PA addition to the cells (Figure S4). Results for PA **4** co-localization with the panel of markers were similar indicating a common internalization pathway for both types of RPARPAR PAs (Figure S5). CTb binds the glycosphingolipid GM1 present in lipid rafts and following internalization it is trafficked through the Golgi to the endoplasmic reticulum [18]. Although CTb is often assumed to represent a specific marker for clathrin-independent uptake, there is evidence that depending on cell type multiple pathways, including clathrin-mediated endocytosis, might be involved in its internalization [18], [19]. Dynamin-2 is necessary for clathrin- and caveolae-mediated endocytosis and we next examined its involvement in PA internalization. Imaging of PA **2** in PPC-1 cells transiently transfected with EGFP-coupled dynamin-2 showed no overlap of PAs with dynamin-2 (Figure 2E). Moreover, PA **2** was still able to internalize in PPC-1 cells transfected with a mutant dynamin-2 dominant negative construct (Dynamin-2(K44A)-EGFP) (Figure 2F). Similar results were obtained with PA **4** (Figure S6). Combined these results suggested that RPARPAR PAs internalized through clathrin-independent carriers (CLICs).

**Figure 3:**
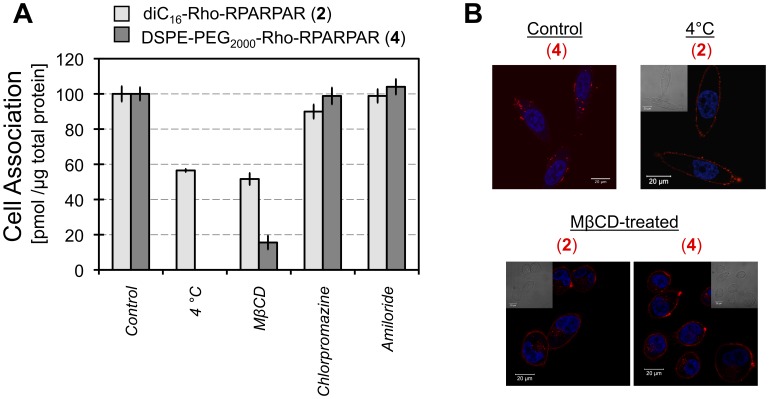
Internalization of PAs requires cholesterol and is not inhibited by chlorpromazine or amiloride. (A) PPC-1 cell association of PA **2** and PA **4** in presence of MβCD (cholesterol depletion agent) was reduced compared to controls Amiloride did not affect cell-association or internalization of PAs, whereas a low (10%) inhibition of cell-association was noted for PA **2** in presence of chlorpromazine. Average values and SEM are presented. (B) Qualitatively, the ratio of PAs localized on the plasma mebrane to the PAs found in intracellular vesicles was higher in MβCD treated-cells indicating that internalization was impaired. PA **2** associated with PPC-1 plasma membrane at 4°C but was not internalized after 1 hour. Scale bars: 20 µm.

CLIC formation and internalization require cholesterol in the plasma membrane [20], [21]. A decrease of 48% in cell association following treatment with the cholesterol-depleting agent methyl-beta-cyclodextrin (MβCD) was found for PA **2** and 84% for PA **4** (Figure 3A). Compared to non-treated cells, MβCD-treated cells exhibited rounded morphology as previously shown [22] but their viability was not affected (Figure 3B). The majority of fluorescence in MβCD-treated cells was associated with the plasma membrane and only a small fraction of membrane-bound PAs was internalized (Figure 3B). The lower cell association of PA **4** with MβCD-treated cells could be due to a lower affinity of this PEG-containing PA for the plasma membrane compared to PA **2**. Despite the similarity in hydrophobic tail and peptide headgroup between the two PAs the presence of the long hydrophilic PEG chain renders PA **4** more hydrophilic. We suggest that PA **4** binds reversibly to the plasma membrane and is removed during the washing step. This hypothesis is also supported by pulse-chase experiments discussed below.

Incubation of PPC-1 cells at 4°C resulted in a 43% decrease in cell association of PA **2 (** Figure 3A). The decrease of association was apparently due to uptake inhibition as demonstrated by confocal microscopy (Figure 3B). PAs were associated with the plasma membrane rather than being internalized indicating that PA internalization is an active process. Inhibition of clathrin mediated endocytosis by chlorpromazine and incubation with amiloride (an inhibitor of Na^+^/H^+^ exchangers and macropinocytosis [23]) did not alter PA cell association compared to the controls (Figure 3A). Collectively, our data suggest CLIC pathway as the major internalization route for PAs in PPC-1 cells.

### PA Trafficking is Determined by the Non-peptidic Part of the Molecule

**Figure 4:**
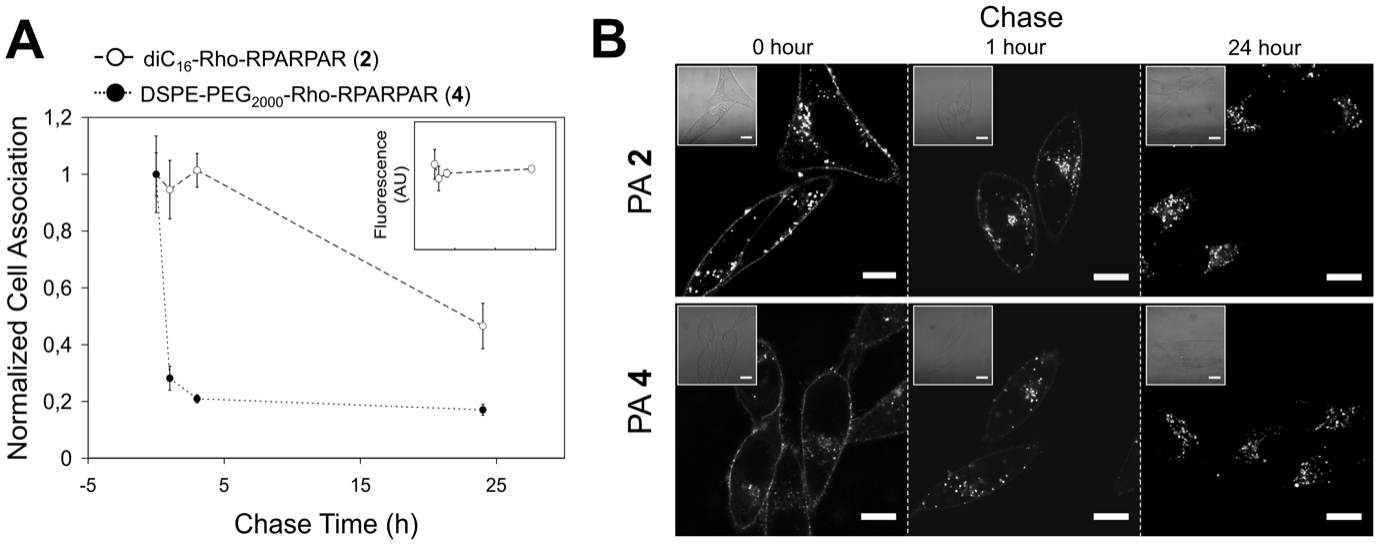
The non-peptidic part determines PA retention in PPC-1 cells. (A) Pulse-chase experiments were performed with 10 µM PAs in PPC-1 cells with 1-hour pulse and different chase periods. Cell association of fluorescent PAs was determined and normalized (value of 1 corresponds to no chase). PPC-1-associated levels of PA **2** remained constant over 3 hours and decreased to half over 24 hours, whereas PA **4** levels decreased to 25% and 20% at 1 and 3 hours, respectively. PA **2** fluorescence values/well remained constant during a 24-hour chase (inset). Average values and standard deviations (n = 3) are presented. (B) Confocal micrographs of the two PAs at different chase points revealed similar intracellular patterns. Scale bars: 20 µM.

We next examined the intracellular fate of PAs and in particular whether they are retained inside cells or are exocytosed with time. Pulse-chase experiments revealed that the amount of PAs inside PPC-1 cells following a 1-hour pulse and different chase periods was dependent on the non-peptidic part (Figure 4A). PA **2** cell association remained unchanged from 1 to 3 hours. At the same time, a loss of approximately 75% in PA **4** cell association occurred within one hour of chase and 80% at 3 hours. After a 24-hour chase, association of PA **2** with cells also decreased by 50%, but this most likely reflects dilution of the label during cell division since the total amount of fluorescence remained constant (Figure 4A-inset).

Confocal micrographs of PAs after different chase times showed qualitatively the same intracellular distribution despite the quantitative differences in cell association (Figure 4B). After 1-hour incubation (chase time = 0), PAs localized in the plasma membrane and in vesicles scattered around the cytoplasm. Chase for 1 hour and 24 hours showed substantial loss of membrane fluorescence and gradual accumulation of PA-containing intracellular vesicles at a perinuclear site (Figure 4B). However, upon closer inspection a difference between the two PAs was observed after a 24-hour chase: whereas PA **4** could be also detected on the plasma membrane, PA **2** was only observed in intracellular vesicles (Figure S7).

**Figure 5:**
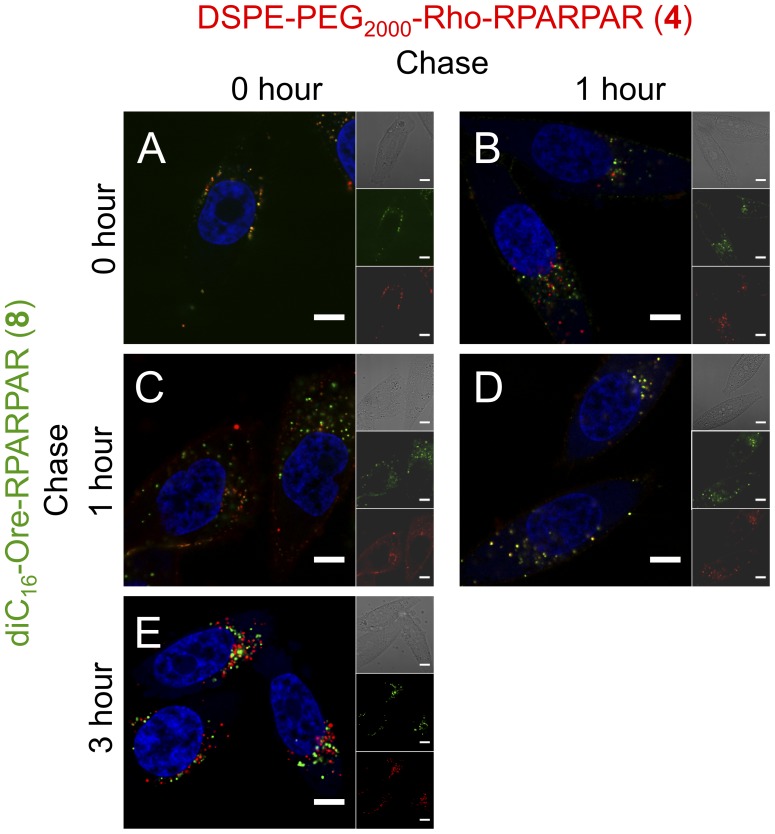
RPARPAR PAs share the same initial internalization pathway and diverge at later time points. Confocal micrographs at different time points of chase (pulse: 1 hour) revealed high extent of co-localization (yellow) between PA **8** (green) and PA **4** (red) when both PAs were chased for the same time (A,D). When PAs were chased for different time points, co-localization extent was decreased and was dependent on which PA was chased (B,C,E and see text for details). Nuclei stain (blue): Hoechst 33342. Scale Bars: 20 µM.

Pulse-chase was next combined with co-localization experiments of PAs **8** (diC_16_ tail) and **4** (DSPE-PEG_2000_ tail) (Figure 5). The two PAs stained the same intracellular vesicles when co-incubated for 1-hour (Figure 5A) as well as when chased for an additional hour (Figure 5D), indicating that they share the same initial internalization and trafficking routes. PPC-1 cells that were pulsed for 1 hour with PA **4**, washed and subsequently incubated for an additional hour with PA **8** did not show overlap of fluorescence demonstrating that PA trafficking after 1 hour has not reached a steady state (Figure 5B). When the order of PA addition was reversed with PA **8** incubated first, some co-localization of PAs was evident (Figure 5C) suggesting that after 1-hour chase PA **8** still resided in intracellular vesicles that PA **4** are trafficked through. When PA **8** chase time was increased to 3 hours, co-localization was reduced indicating that the two PAs co-localized transiently (Figure 5E).

**Figure 6:**
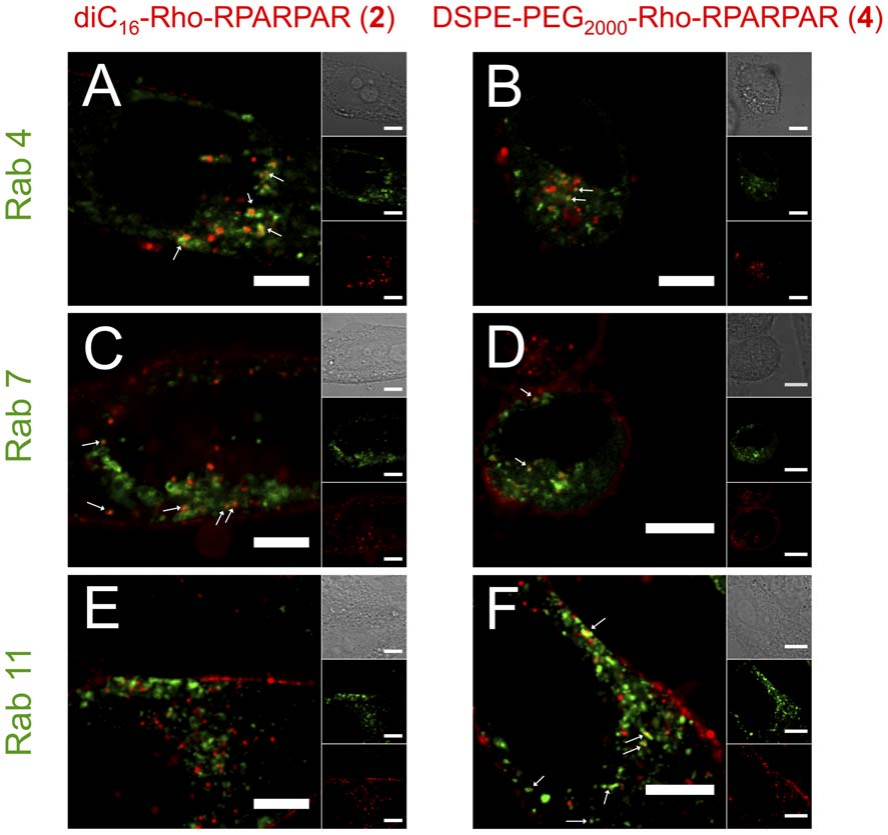
RPARPAR PAs are recycled to the plasma membrane and trafficked to late endosomes. Confocal micrographs of PPC-1 cells transfected with different EGFP-coupled Rab family members and incubated for 1 hour with 10 µM RPARPAR PAs. Both PAs (**2** and **4**) co-localized with Rab4-positive vesicles (A, B) and Rab7-positive vesicles (C, D). PA **4** additionally stained Rab11-positive vesicles (F). Arrows indicate examples of co-localization. Scale bars: 20 µM.

**Figure 7:**
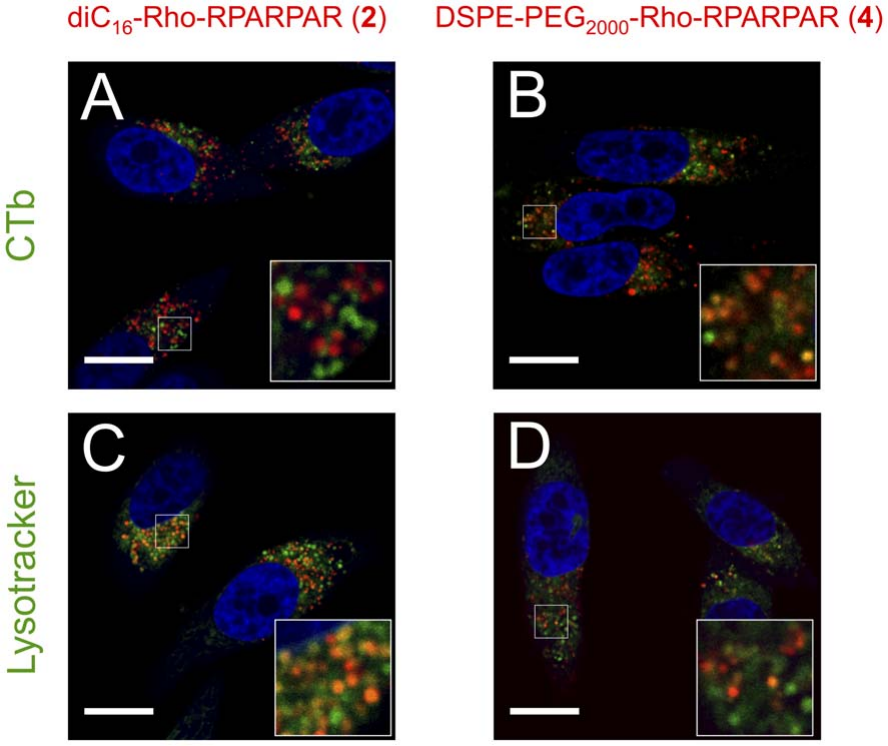
diC_16_-Rho-RPARPAR (2) is eventually trafficked to lysosomes while DSPE-PEG_2000_-Rho-RPARPAR (4) is additionally found in CTb-positive vesicles after 24 hour incubation. Confocal micrographs or RPARPAR PAs following a 1-hour pulse and 24-hour chase in PPC-1 cells treated either with CTb for 1 hour (A, B) or stained with lysotracker (C, D) showed differences in PA localization. PA **2** co-localized with lysosomes (C) but not CTb (A). PA **4** on the other hand, co-localized with both CTb (B) and lysosomes (D). Scale bars: 20 µm.

We hypothesized that quantitative differences in PA retention observed in the pulse-chase experiments were due to a combination of altered PA trafficking and differences in membrane affinity of the two PAs. We postulated that both PAs are recycled to the plasma membrane, where the more hydrophilic PA **4** would be washed away whereas PA **8 (**or **2**) would be firmly anchored to the plasma membrane and thus be re-internalized. This hypothesis is consistent with a reduction of PA **4** intracellular levels with time. It also explains why higher co-localization was observed when PA **8** was chased for 1 hour and PA **4** was pulsed for 1 hour compared to when the order was reversed. To test our hypothesis, we transiently transfected PPC-1 cells with Rab4, Rab7, and Rab11 and studied the co-localization of the PAs with Rab-positive vesicles [24], [25] (Figure 6). Both PA **2** and **4** co-localized with Rab4-positive vesicles confirming that a fraction of internalized PAs is recycled via the ‘short-loop’ recycling route to the plasma membrane (Figure 6A&B). PA **4** additionally showed low levels of co-localization with Rab11-positive vesicles suggesting that recycling occurred also via the ‘long-loop’ recycling (Figure 6F). PA **2** on the other hand did not co-localize with Rab11 (Figure 6E). Both PAs co-localized with the late-endosomal marker Rab7 indicating that they are trafficked towards lysosomes (Figure 6C&D) even though they had not reached these organelles after 1 hour (Figures 1&S5). After a 24-hour chase, extensive co-localization of PAs with lysosomes was observed (Figure 7). However, whereas nearly all PA **2**-positive vesicles were identified as lysosomes, PA **4** showed substantial co-localization with CTb-positive vesicles. Collectively, these results show that differences in PA chemical structure lead to differences in their intracellular trafficking.

## Discussion

Our data show that PAs associate with cells *in vitro* by virtue of their hydrophobic tail and subsequent internalization and trafficking are dependent on the chemical structure of the tail and linker to the peptide. RPARPAR PAs did not rely on the CendR mechanism for cell association since *i)* RPARPAR PAs with amidated (PAs **3**&**5)** and carboxylated (PAs **2**&**4)** C-terminus were associated with PPC-1 cells at the same levels [4] and *ii)* high PA association was observed with a cell line lacking the CendR receptor NRP1. Nevertheless, the peptide sequence significantly influenced cell association of PAs. PA **2** association was higher than that of PA **7** (diC_16_ tails) and PA **4** was associated to a greater extent than amphiphile **6** (DSPE-PEG_2000_ tails). It is not yet clear whether the peptide headgroup dependence was an effect of altered PA hydrophilicity, a consequence of non-specific cell binding or a combination of both. Arginine-rich peptides are known to bind strongly to the cell membrane and subsequently internalize into cells, with many CPPs based on arginine [3], [26]. At the same time, the higher hydrophilicity of the charged peptide headgroup is expected to increase its desorption rate in an aqueous environment [27], thus accelerating PA transfer between plasma membrane, proteins and self-assembled objects [10], [28].

A CLIC internalization pathway of RPARPAR PAs following their anchoring to the plasma membrane was common for the two different tested lipid tails. RPARPAR PAs co-localized after 1-hour pulse and 1-hour chase and exhibited identical staining patterns with intracellular markers. The saturated diC_16_- and DSPE- tails of both PAs used in this study are expected to associate with cholesterol-rich, ordered-lipid domains known as detergent-resistant membranes or lipid rafts [29]. Co-localization with cholera toxin subunit B (CTb) supported this prediction. Cholesterol depletion significantly inhibited internalization of both RPARPAR PAs; interestingly however, cholesterol depletion did not abolish PA binding to the plasma membrane of MβCD-treated cells (Figure 3), indicating its role in vesicle budding and not in promoting membrane anchoring. Clathrin-mediated endocytosis was not a major uptake mechanism of RPARPAR PAs, since their internalization was independent of dynamin and not inhibited by chlorpromazine; however, we cannot exclude some minor contribution of this pathway.

Structurally related amphiphilic lipid-like molecules have previously been shown to employ CLIC pathways, often in combination with clathrin-mediated uptake (CME). A CLIC pathway is employed by the naturally occurring, membrane bound glycophosphatidylinositol (GPI) proteins, which position on the exoplasmic leaflet of the membrane via a glycolipid anchor [21], [30]. Synthetic analogs, including a fluorescent lipid molecule and a PEG-lipid conjugate co-localized with both transferrin (marker for CME) and folate (marker for CLIC pathway); a slight preference for the PEG-lipid to internalize by CLIC pathways was noted [31]. Finally, the diC_16_p53 PA used in this study as a control was previously shown to mainly utilize CME for cell entry in SJSA-1 osteosarcoma cells [15]. Therefore, it appears that PAs and similar amphiphiles do not necessarily utilize identical internalization pathways, depending on the cell type and nature of the hydrophilic headgroup.

The fate of PAs following uptake is important for intracellular targeting applications. Our findings demonstrate that the chemical structure of the non-peptidic part is responsible for dissimilar trafficking of the two RPARPAR PAs studied. PA **4** was present in Rab4 and Rab11 positive vesicles suggesting that it is recycled to the membrane using both the short and long recycling loops [24], [25]. After 24 hours, a small fraction of this PA was still found in the plasma membrane while most of it was localized in lysosomes and endocytic vesicles, in which CTb accumulates after 1-hour incubation. On the other hand, PA **2** co-localized predominantly with Rab4-positive vesicles at early time points and at 24 hours the majority of the PA was found in lysosomes with plasma membrane devoid of it.

**Figure 8:**
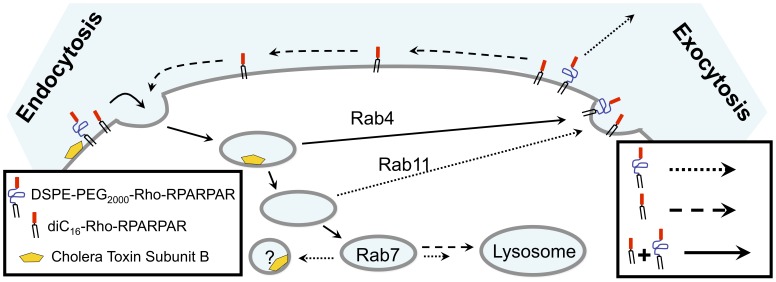
Proposed model for internalization and trafficking of RPARPAR PAs. Both PAs bind the plasma membrane and are taken up primarily via clathrin-independent pathways (solid line). Both PAs are recycled to the plasma membrane but diC_16_ PAs remains anchored to it (dashed line), whereas DSPE-PEG_2000_ PAs is washed away (dotted line). The diC_16_ tail is directed to lysosomes while DSPE-PEG_2000_ is trafficked both to lysosomes and CTb-containing organelles.

We have summarized our results in a hypothetical model for PA trafficking in Figure 8. Both types of RPARPAR PA utilize the same routes for cell entry and co-localize with Rab4, a marker for ‘short loop’ recycling endosomes. However, diversion of PA **4** from the lysosomes towards the Rab11-dependent recycling to the plasma membrane results in dissimilar trafficking at longer time points. The increased hydrophilicity and consequent increased desorption rate is responsible for the decrease in its cell-association during chase experiments: each time the PA undergoes an exocytosis-endocytosis cycle some of it is desorbed from the plasma membrane.

The idea that trafficking of lipid-like amphiphiles is determined by the chemistry of their hydrophobic tails was first proposed by Mukherjee *et al*. [32]. The authors proposed that distribution of amphiphilic dyes in membrane regions of differing fluidity determine their localization in sorting endosomes and therefore determine their ensuing trafficking. In our case, the difference in 2 methylene units/acyl chain is not expected to significantly influence the PA membrane distribution since both PAs partition in lipid-ordered domains at physiological temperature and pH [29], [33]. On the other hand, the contrast in headgroup size and steric effects might be the determining factor. This argument, introduced by Bhagatji *et al.* for explaining how GPI-anchored proteins are excluded from protein-dense clathrin-coated pits [31] could also influence sorting in recycling endosomes.

The findings of our study provide important considerations on peptide amphiphile design. The reversible cell association of the PEG-containing PA is expected to enhance their tissue penetration: internalized PAs are not trapped in the first cell they encounter but can exit and diffuse to neighboring cells. Another potential advantage of the PEG-containing lipids is their routing away of the lysosomes. Avoiding the harsh environment of these organelles should protect peptide drugs from degradation. On the other hand, the lower stability of PEG-lipids supramolecular constructs compared to the shorter, more hydrophobic, ‘classical’ PAs raises concerns for their in vivo stability and biodistrubution. Future work should focus on *in vivo* tumor targeting and tissue penetration of the two different PA architectures and correlate the findings to the *in vitro* results described here. In conclusion, we have shown that PA architecture is a way to control intracellular trafficking and retention of a model peptide.

## Materials and Methods

### Peptides & Peptide Amphiphiles (PAs)

Peptides were synthesized using standard solid phase synthesis methods either on a Rink Amide resin or a Wang resin. diC_16_RPARPAR PAs were synthesized on the resin by coupling the di-alkyl lipid acid 4-(1,5-bis(hexadecyloxy)-1,5-dioxopentan-2-ylamino)-4-oxobutanoic (diC_16_) [17] on peptide’s N-terminus. DSPE-PEG_2000_-RPARPAR PAs were synthesized by coupling RPAR peptides bearing a cysteine to 1,2-distearoyl-*sn*-glycero-3-phosphoethanolamine-N-maleimide(polyethylene glycol)_2000_ (Avanti Polar Lipids) at 1∶1 molar ratio in PBS 10 mM at room temperature for 1 hour. Peptides and PAs were purified using high-performance liquid chromatography (HPLC; Shimadzu Corporation) and identity was verified using matrix-assisted laser desorption/ionization (MALDI) time-of-flight (TOF) mass spectrometry. Materials of purity greater than 95% were stored dry at −20°C until used. A list of the peptides and PAs used is presented in Figure 1. PA solutions were prepared as follows: PAs were dissolved in a 1∶1 mixture of chloroform and methanol and solvents were evaporated under N_2_ flow to form a PA film on a glass vial, which was then dried in vacuum. PA films were then hydrated at 60°C for 1 hour. PA solutions were stored at 4°C and used within 1 week of preparation.

### Cell Culture

PPC-1 and M21 cell lines [4] were cultured as exponentially growing, sub-confluent monolayers in DMEM cell culture medium (ATCC) supplemented with 10% v/v calf bovine serum (ATCC) and 0.1% v/v Penicillin/Streptomycin (Gibco). Cells were grown at 37°C, humidified atmosphere and 5% CO_2_. Cells were detached following a 5-minute incubation with a 2 mM EDTA solution in PBS. For all *in vitro* studies PPC-1 and M21 cells were seeded at a density of 2×10^4^ cells/cm^2^. Typically cells were allowed to attach on the surfaces overnight (12–16 h) prior to experiments.

### Cell Association Studies

Association of peptides and PAs with adherent cells was determined using fluorescence spectroscopy measurements at 37°C, unless otherwise noted. Cells seeded in 12-well plates were incubated with fluorescent peptides or PAs, washed once with PBS 10 mM, detached and placed in 15 ml centrifuge tubes. Cells were centrifuged for 5 minutes at 1400 rpm, supernatant was discarded and 2 ml PBS 10 mM was used to resuspend the cells. Following a second centrifugation and removal of supernatant, 0.5 ml of triton X-100 in water (1% v/v) were added and the solutions briefly vortexed to lyse the cells. Three 100 µl aliquots were used to measure fluorescence intensity using a Tecan Infinite M200 and two 50 µl aliquots were used with the BCA protein assay kit in order to calculate total protein content. Fluorescence intensity was converted to peptide/PA concentration using calibration curves constructed with pure material in triton X-100 aqueous solutions.

### Pulse-Chase Experiments

In pulse-chase experiments, cells were incubated for a first time period (pulse) with a PA, washed with PBS and subsequently incubated for a second time period (chase) in cell culture medium or in presence of a different PA.

### Imaging

For microscopy studies, cells were seeded in Lab-Tek chambered coverglass slides (Nalge Nunc). Following incubation with formulations under set conditions cells were washed two times with sterile filtered PBS (10 mM, pH 7.4). In some cases, Hoechst 33342 was added 10 minutes prior to washing to stain cell nuclei. For live cell imaging, cells were visualized in supplemented cell culture medium using a Nikon Eclipse TE-200 microscope equipped with a 10x and 100x objective and a 100 W mercury arc lamp. Images were processed and false color was added using ImageJ software. Alternatively, cells were fixed using 4% parafolmadehyde solution in phosphate buffer (100 mM) for 30 minutes at room temperature. After fixation, cells were washed and imaged in PBS using a Zeiss Laser Scanning Confocal microscope (LSM 700) equipped with 20x and 63x objectives and 405, 488 and 555 nm solid-state lasers for fluorophore excitation. Image processing was performed using the Zen software provided by Zeiss.

### Inhibition, Co-localization and Transfection Studies

A number of different conditions were used in order to gain insight on cell internalization mechanisms. Cells were incubated with 10 µg/ml chlorpromazine or 1 mM amiloride or 5 mM methyl-β-cyclodextrin (MβCD) or in absence of serum and antibiotics, 30 minutes prior to addition of PAs. Inhibitors were present also during PA incubation. Cell association was performed at 4°C to assess energy requirements. Co-localization studies were performed by incubating cells with PAs and 1) 0.36 nM cholera toxin subunit B, alexa fluor 488 conjugate (Invitrogen), 2) 100 nM Mitotracker Deep Red (Invitrogen), 3) 20 µM Lysotracker Green (Invitrogen) and 4) 10 µM fluorescein-labeled human transferrin. PPC-1 cells were transfected using Fugene HD transfection agent with the following plasmids (kind gifts from prof. Dzwokai Ma, UCSB): 1) Rab4b-EGFP, 2) Rab7-EGFP, 3) Rab11-EGFP, 4) Dynamin-2-EGFP and 5) mutant Dynamin-2(K44A)-EGFP (EGFP: Enhanced Green Fluorescent Protein). Briefly, 10 µl of Fugene transfection agent was mixed with 2 µg plasmid/100 µl DMEM and incubated at room temperature for 10 minutes. The complexes were then added to PPC-1 cells and incubated for 24 hours in cell culture medium without Pen/Strep. Cells were washed twice with PBS 10 mM before incubation with PAs.

## Supporting Information

Figure S1 **diC_16_-Rho-RPARPAR (2) incorporates in the plasma membrane within 1 minute of incubation.** Confocal micrographs of PPC-1 cells incubated with 10 µM PA **2** for 1 and 10 minutes. Scale bars: 20 μΜ.
(TIFF)

Figure S2 **Intracellular localization of amphiphiles.** Confocal micrographs (not normalized in respect to fluorescence intensity) of cell-associated PAs **3**, **4** and **8** and control amphiphile **6** (concentration: 10 µM) after 1-hour incubation with PPC-1 cells. A similar intracellular fluorescence pattern for all amphiphiles was noted, independent of tail and fluorescence label. Nuclei stain (blue): Hoechst 33342; Scale bars: 40 µm.
(TIFF)

Figure S3 **Live cell epifluorescence microscopy of PPC-1 and M21 cells incubated for 1 hour with 10** **µM diC_16_-Rho-RPARPAR (2).** Intracellular fluorescence distribution was similar to that observed in fixed cells, indicating the absence of fixation artifacts. Nuclei stain (blue): Hoechst 33342. Scale bars: 10 µm.
(TIFF)

Figure S4 **Early colocalization of PA 2 with CTb.** Confocal micrograph of PPC-1 cells incubated with cholera toxin subunit B (20 µg/ml) for 1 hour and 10 µM diC_16_-Rho-RPARPAR (**2)** for 10 minutes. The majority of PA **2** co-localized with CTb in intracellular vesicles (arrows); however, a few PA **2**-positive only vesicles were observed (arrowheds). Scale bars: 20 µm.
(TIFF)

Figure S5 **Co-localization of PA 4 with intracellular markers.** PPC-1 cells incubated with 10 µM DSPE-PEG_2000_-Rho-RPARPAR (**4**) were co-localized with CTb (green; yellow indicates co-localization) but not with mitochondria (Mitotracker; green) or lysosomes (Lysotracker; green). A small fraction of intracellular vesicles were positive for both PA **4** and transferrin. Blue: Hoechst 33342; Scale bars: 10 µm.
(TIFF)

Figure S6 **DSPE-PEG_2000_-Rho-RPARPAR (4) enters cells in a dynamin-2-independent manner.** PPC-1 cells were transfected with EGFP-coupled dynamin-2 (A) or a dominant negative dynamin-2 mutant (B). 24 hours after transfection, cells were incubated for 1 hour with 10 µM PA **4**. Absence of co-localization with dynamin-2 (A) and internalization in PPC-1 cells expressing the dominant negative dynamin-2 mutant (B) indicate that PA **4** does not require dynamin-2 for internalization. Nuclear Stain (Blue): Hoechst 33342; Scale bars: 20 µm.
(TIFF)

Figure S7 **Membrane association after 24 h depends on PA architecture.** PPC-1 cells pulsed 1 hour with diC_16_-Rho-RPARPAR (**2**) or DSPE-PEG_2000_-Rho-RPARPAR (**4**) and chased for 24 hours. A fraction of PA **4** was present on plasma membranes; in contrast, no PA **2** was detected on the plasma membrane. Confocal micrographs were processed to highlight membrane presence (or absence) of PAs.
(TIFF)

## References

[1] Ruoslahti E, Bhatia SN, Sailor MJ (2010) Targeting of drugs and nanoparticles to tumors. The Journal of Cell Biology 188: 759–768 doi:10.1083/jcb.200910104. 20231381 10.1083/jcb.200910104 PMC2845077

[2] Teesalu T, Sugahara KN, Ruoslahti E (2012) Mapping of vascular ZIP codes by phage display. Meth Enzymol 503: 35–56 doi:10.1016/B978-0-12-396962-0.00002-1. 22230564 10.1016/B978-0-12-396962-0.00002-1

[3] Foerg C, Merkle HP (2007) On the biomedical promise of cell penetrating peptides: Limits versus prospects. J Pharm Sci 97: 144–162 doi:10.1002/jps.21117. 10.1002/jps.21117 17763452

[4] Teesalu T, Sugahara KN, Kotamraju VR, Ruoslahti E (2009) C-end rule peptides mediate neuropilin-1-dependent cell, vascular, and tissue penetration. Proceedings of the National Academy of Sciences 106: 16157–16162 doi:10.1073/pnas.0908201106. 10.1073/pnas.0908201106 PMC2752543 19805273

[5] Roth L, Agemy L, Kotamraju VR, Braun G, Teesalu T, et al.. (2011) Transtumoral targeting enabled by a novel neuropilin-binding peptide. Oncogene. doi:10.1038/onc.2011.537. 10.1038/onc.2011.537 22179825

[6] Cui H, Webber MJ, Stupp SI (2010) Self-assembly of peptide amphiphiles: From molecules to nanostructures to biomaterials. Biopolymers 94: 1–18 doi:10.1002/bip.21328. 20091874 10.1002/bip.21328 PMC2921868

[7] Lin BF, Marullo RS, Robb MJ, Krogstad DV, Antoni P, et al (2011) De Novo Design of Bioactive Protein-Resembling Nanospheres via Dendrimer-Templated Peptide Amphiphile Assembly. Nano Lett 11: 3946–3950 doi:10.1021/nl202220q. 21800917 10.1021/nl202220q PMC3223106

[8] Braun GB, Pallaoro A, Wu G, Missirlis D, Zasadzinski JA, et al (2009) Laser-Activated Gene Silencing viaGold Nanoshell−siRNA Conjugates. ACS Nano 3: 2007–2015 doi:10.1021/nn900469q. 19527019 10.1021/nn900469q

[9] Trent A, Marullo R, Lin B, Black M, Tirrell M (2011) Structural properties of soluble peptide amphiphile micelles. Soft Matter 7: 9572 doi:10.1039/c1sm05862b.

[10] Kastantin M, Ananthanarayanan B, Karmali P, Ruoslahti E, Tirrell M (2009) Effect of the Lipid Chain Melting Transition on the Stability of DSPE-PEG(2000) Micelles. Langmuir 25: 7279–7286 doi:10.1021/la900310k. 19358585 10.1021/la900310k PMC2756452

[11] Peters D, Kastantin M, Kotamraju VR, Karmali PP, Gujraty K, et al (2009) Targeting atherosclerosis by using modular, multifunctional micelles. Proceedings of the National Academy of Sciences 106: 9815–9819 doi:10.1073/pnas.0903369106. 10.1073/pnas.0903369106 PMC2689312 19487682

[12] Karmali PP, Kotamraju VR, Kastantin M, Black M, Missirlis D, et al (2009) Targeting of albumin-embedded paclitaxel nanoparticles to tumors. Nanomedicine 5: 73–82 doi:10.1016/j.nano.2008.07.007. 18829396 10.1016/j.nano.2008.07.007 PMC2824435

[13] Sugahara KN, Teesalu T, Karmali PP, Kotamraju VR, Agemy L, et al (2009) Tissue-Penetrating Delivery of Compounds and Nanoparticles into Tumors. Cancer Cell 16: 510–520 doi:10.1016/j.ccr.2009.10.013. 19962669 10.1016/j.ccr.2009.10.013 PMC2791543

[14] Kastantin M, Missirlis D, Black M, Ananthanarayanan B, Peters D, et al (2010) Thermodynamic and Kinetic Stability of DSPE-PEG(2000) Micelles in the Presence of Bovine Serum Albumin. J Phys Chem B 114: 12632–12640 doi:10.1021/jp1001786. 20828210 10.1021/jp1001786

[15] Missirlis D, Krogstad DV, Tirrell M (2010) Internalization of p53 14−29Peptide Amphiphiles and Subsequent Endosomal Disruption Results in SJSA-1 Cell Death. Mol Pharmaceutics 7: 2173–2184 doi:10.1021/mp100193h. 10.1021/mp100193h PMC2997927 20822110

[16] Missirlis D, Khant H, Tirrell M (2009) Mechanisms of Peptide Amphiphile Internalization by SJSA-1 Cells in Vitro†. Biochemistry 48: 3304–3314 doi:10.1021/bi802356k. 19245247 10.1021/bi802356k PMC2713106

[17] Berndt P, Fields GB, Tirrell M (1995) Synthetic lipidation of peptides and amino acids: monolayer structure and properties. JACS 117: 9515–9522.

[18] Chinnapen DJF, Chinnapen H, Saslowsky D, Lencer WI (2007) Rafting with cholera toxin: endocytosis and trafficking from plasma membrane to ER. FEMS Microbiology Letters 266: 129–137 doi:10.1111/j.1574-6968.2006.00545.x. 17156122 10.1111/j.1574-6968.2006.00545.x PMC3511785

[19] Torgersen ML, Skretting G, van Deurs B, Sandvig K (2001) Internalization of cholera toxin by different endocytic mechanisms. Journal of Cell Science 114: 3737–3747. 11707525 10.1242/jcs.114.20.3737

[20] Hansen CG, Nichols BJ (2009) Molecular mechanisms of clathrin-independent endocytosis. Journal of Cell Science 122: 1713–1721 doi:10.1242/jcs.033951. 19461071 10.1242/jcs.033951 PMC2723140

[21] Sabharanjak S, Sharma P, Parton RG, Mayor S (2002) GPI-anchored proteins are delivered to recycling endosomes via a distinct cdc42-regulated, clathrin-independent pinocytic pathway. Developmental Cell 2: 411–423. 11970892 10.1016/s1534-5807(02)00145-4

[22] Ramprasad OG, Srinivas G, Rao KS, Joshi P, Thiery JP, et al (2007) Changes in cholesterol levels in the plasma membrane modulate cell signaling and regulate cell adhesion and migration on fibronectin. Cell Motil Cytoskeleton 64: 199–216 doi:10.1002/cm.20176. 17238130 10.1002/cm.20176

[23] West MA, Bretscher MS, Watts C (1989) Distinct endocytotic pathways in epidermal growth factor-stimulated human carcinoma A431 cells. J Cell Biol 109: 2731–2739. 2556406 10.1083/jcb.109.6.2731 PMC2115909

[24] Zerial M, McBride H (2001) Rab proteins as membrane organizers. Nat Rev Mol Cell Biol 2: 107–117 doi:10.1038/35052055. 11252952 10.1038/35052055

[25] Stenmark H (2009) Rab GTPases as coordinators of vesicle traffic. Nat Rev Mol Cell Biol 10: 513–525 doi:10.1038/nrm2728. 19603039 10.1038/nrm2728

[26] Schmidt N, Mishra A, Lai GH, Wong GCL (2010) Arginine-rich cell-penetrating peptides. FEBS Letters 584: 1806–1813 doi:10.1016/j.febslet.2009.11.046. 19925791 10.1016/j.febslet.2009.11.046

[27] Silvius JR, Zuckermann MJ (1993) Interbilayer transfer of phospholipid-anchored macromolecules via monomer diffusion. Biochemistry 32: 3153–3161 doi:10.1021/bi00063a030. 7681327 10.1021/bi00063a030

[28] Shahinian S, Silvius JR (1995) Doubly-lipid-modified protein sequence motifs exhibit long-lived anchorage to lipid bilayer membranes. Biochemistry 34: 3813–3822 doi:10.1021/bi00011a039. 7893678 10.1021/bi00011a039

[29] Zacharias DA, Violin JD, Newton AC, Tsien RY (2002) Partitioning of lipid-modified monomeric GFPs into membrane microdomains of live cells. Science 296: 913–916 doi:10.1126/science.1068539. 11988576 10.1126/science.1068539

[30] Chatterjee S, Mayor S (2001) The GPI-anchor and protein sorting. Cell Mol Life Sci 58: 1969–1987. 11814051 10.1007/PL00000831 PMC11337326

[31] Bhagatji P, Leventis R, Comeau J, Refaei M, Silvius JR (2009) Steric and not structure-specific factors dictate the endocytic mechanism of glycosylphosphatidylinositol-anchored proteins. The Journal of Cell Biology 186: 615–628 doi:10.1083/jcb.200903102. 19687251 10.1083/jcb.200903102 PMC2733760

[32] Mukherjee S, Soe TT, Maxfield FR (1999) Endocytic sorting of lipid analogues differing solely in the chemistry of their hydrophobic tails. J Cell Biol 144: 1271–1284. 10087269 10.1083/jcb.144.6.1271 PMC2150570

[33] Wang T-Y, Leventis R, Silvius JR (2005) Artificially lipid-anchored proteins can elicit clustering-induced intracellular signaling events in Jurkat T-lymphocytes independent of lipid raft association. J Biol Chem 280: 22839–22846 doi:10.1074/jbc.M502920200. 15817446 10.1074/jbc.M502920200

