# Supplementary figures and images for: The Non-Peptidic Part Determines the Internalization Mechanism and Intracellular Trafficking of Peptide Amphiphiles
Source: PLoS One. 2013 Jan 17;8(1):e54611. doi: 10.1371/journal.pone.0054611 (PMC3547919; doi:10.1371/journal.pone.0054611)

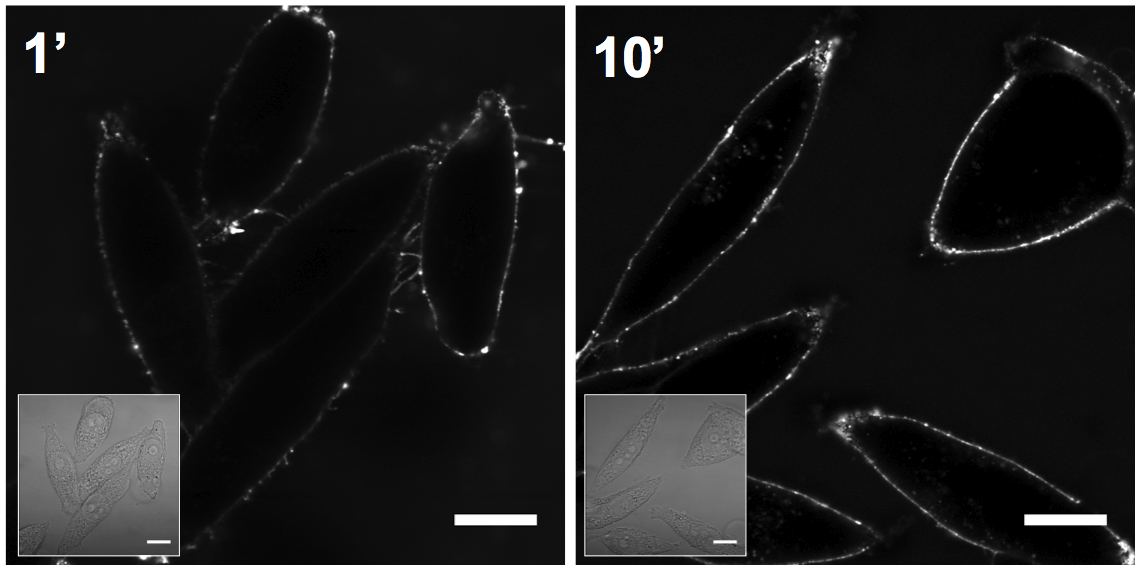

Supplement: Figure S1 — diC16-Rho-RPARPAR (2) incorporates in the plasma membrane within 1 minute of incubation. Confocal micrographs of PPC-1 cells incubated with 10 µM PA 2 for 1 and 10 minutes. Scale bars: 20 μΜ. (TIFF) [file pone.0054611.s001.tiff]

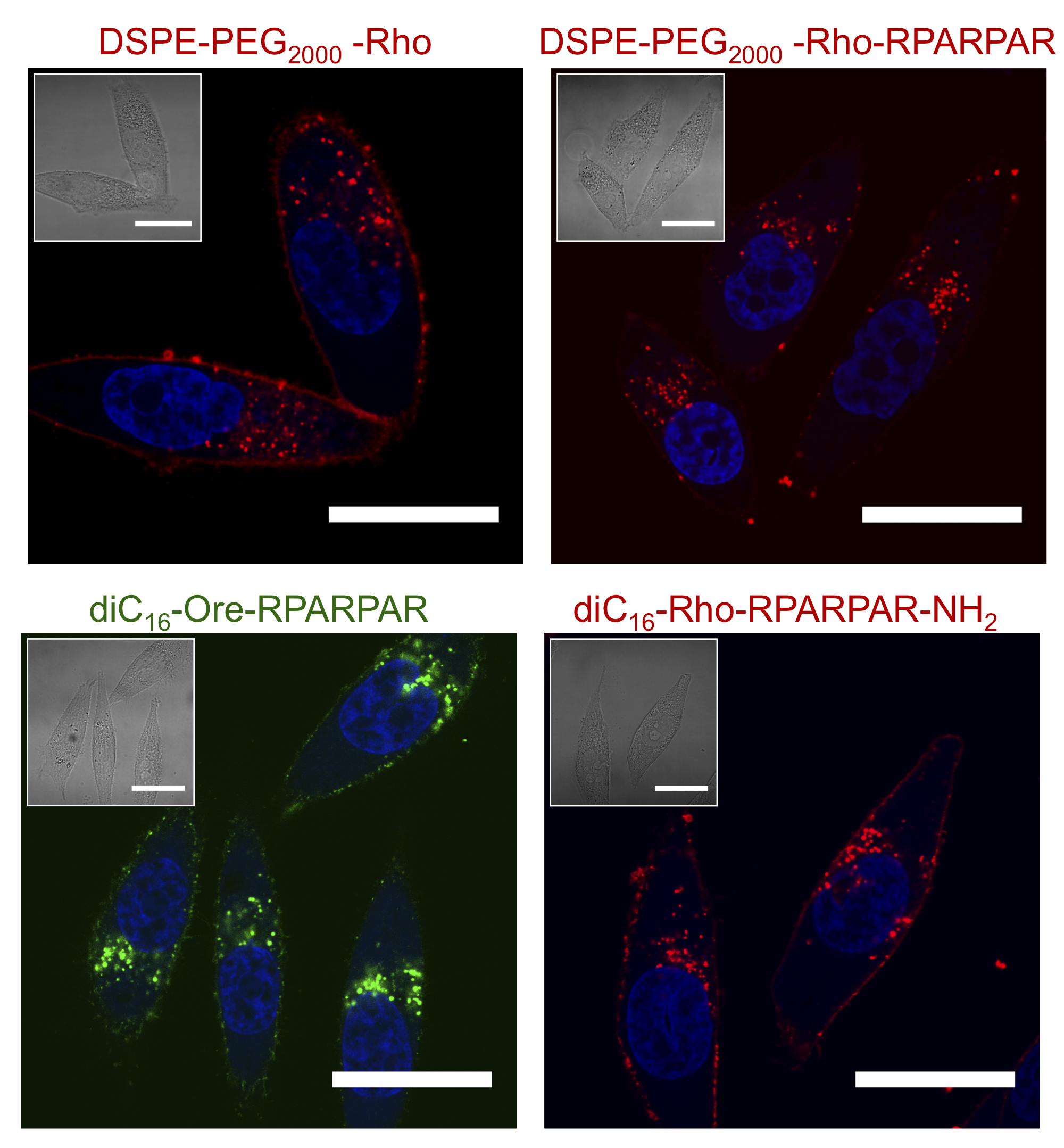

Supplement: Figure S2 — Intracellular localization of amphiphiles. Confocal micrographs (not normalized in respect to fluorescence intensity) of cell-associated PAs 3, 4 and 8 and control amphiphile 6 (concentration: 10 µM) after 1-hour incubation with PPC-1 cells. A similar intracellular fluorescence pattern for all amphiphiles was noted, independent of tail and fluorescence label. Nuclei stain (blue): Hoechst 33342; Scale bars: 40 µm. (TIFF) [file pone.0054611.s002.tiff]

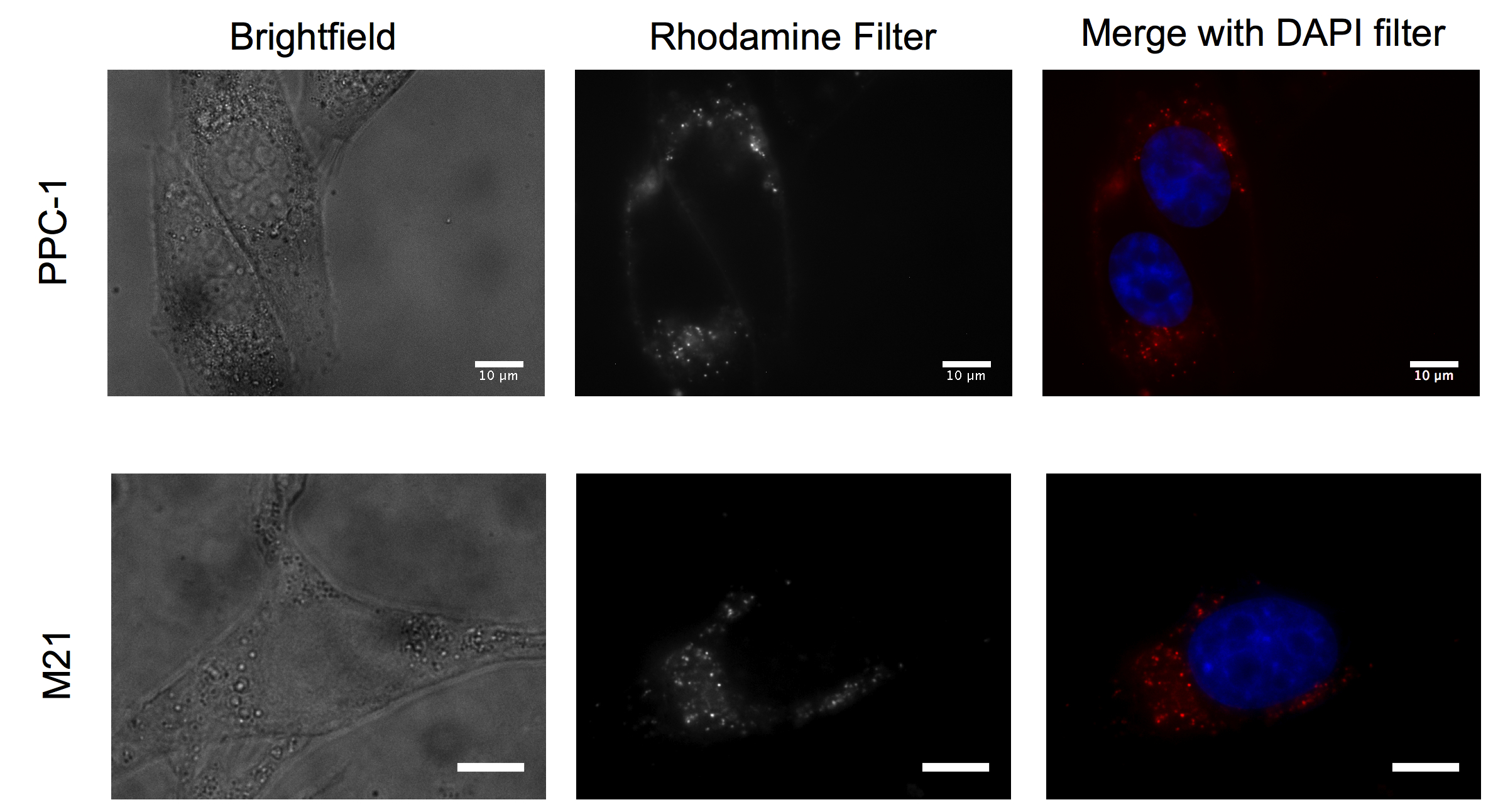

Supplement: Figure S3 — Live cell epifluorescence microscopy of PPC-1 and M21 cells incubated for 1 hour with 10 µM diC16-Rho-RPARPAR (2). Intracellular fluorescence distribution was similar to that observed in fixed cells, indicating the absence of fixation artifacts. Nuclei stain (blue): Hoechst 33342. Scale bars: 10 µm. (TIFF) [file pone.0054611.s003.tiff]

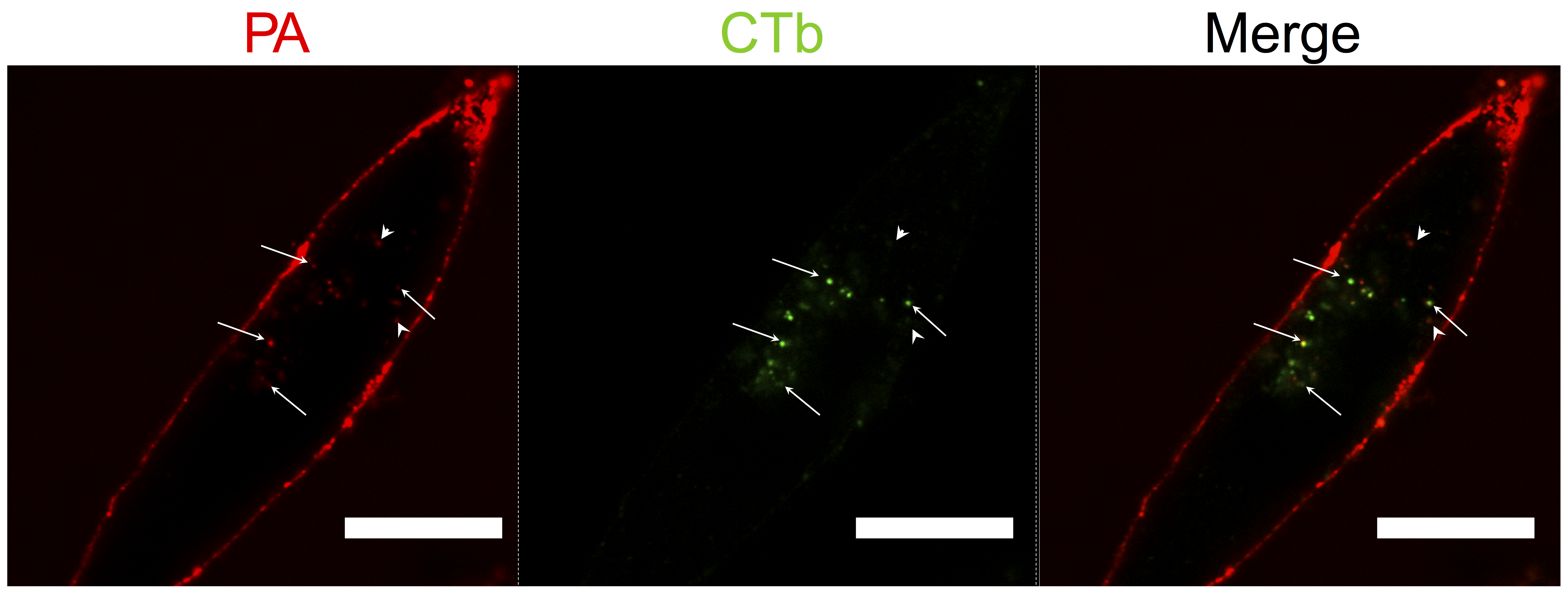

Supplement: Figure S4 — Early colocalization of PA 2 with CTb. Confocal micrograph of PPC-1 cells incubated with cholera toxin subunit B (20 µg/ml) for 1 hour and 10 µM diC16-Rho-RPARPAR (2) for 10 minutes. The majority of PA 2 co-localized with CTb in intracellular vesicles (arrows); however, a few PA 2-positive only vesicles were observed (arrowheds). Scale bars: 20 µm. (TIFF) [file pone.0054611.s004.tiff]

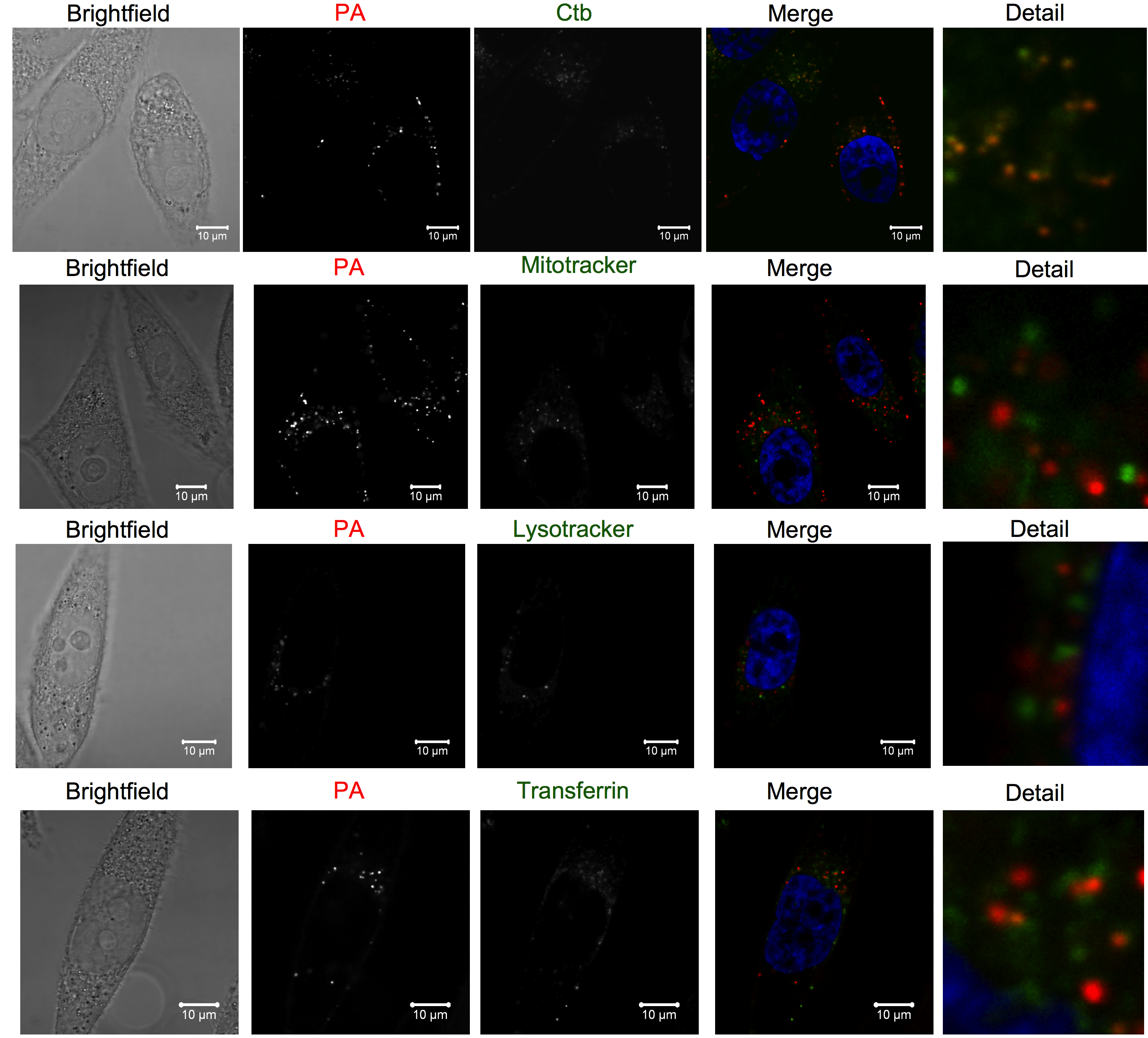

Supplement: Figure S5 — Co-localization of PA 4 with intracellular markers. PPC-1 cells incubated with 10 µM DSPE-PEG2000-Rho-RPARPAR (4) were co-localized with CTb (green; yellow indicates co-localization) but not with mitochondria (Mitotracker; green) or lysosomes (Lysotracker; green). A small fraction of intracellular vesicles were positive for both PA 4 and transferrin. Blue: Hoechst 33342; Scale bars: 10 µm. (TIFF) [file pone.0054611.s005.tiff]

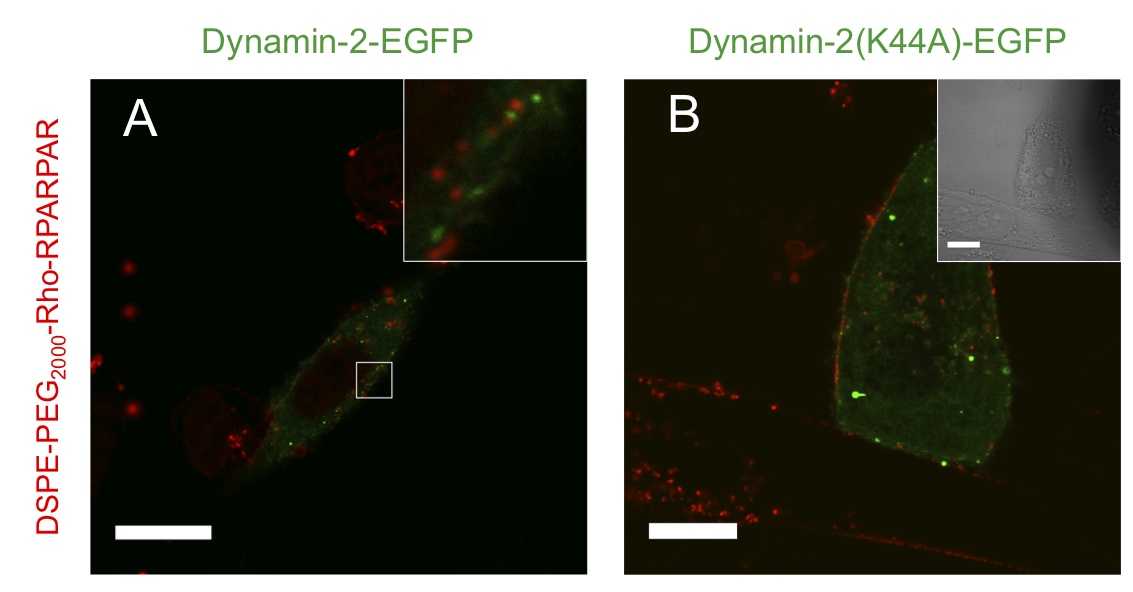

Supplement: Figure S6 — DSPE-PEG2000-Rho-RPARPAR (4) enters cells in a dynamin-2-independent manner. PPC-1 cells were transfected with EGFP-coupled dynamin-2 (A) or a dominant negative dynamin-2 mutant (B). 24 hours after transfection, cells were incubated for 1 hour with 10 µM PA 4. Absence of co-localization with dynamin-2 (A) and internalization in PPC-1 cells expressing the dominant negative dynamin-2 mutant (B) indicate that PA 4 does not require dynamin-2 for internalization. Nuclear Stain (Blue): Hoechst 33342; Scale bars: 20 µm. (TIFF) [file pone.0054611.s006.tiff]

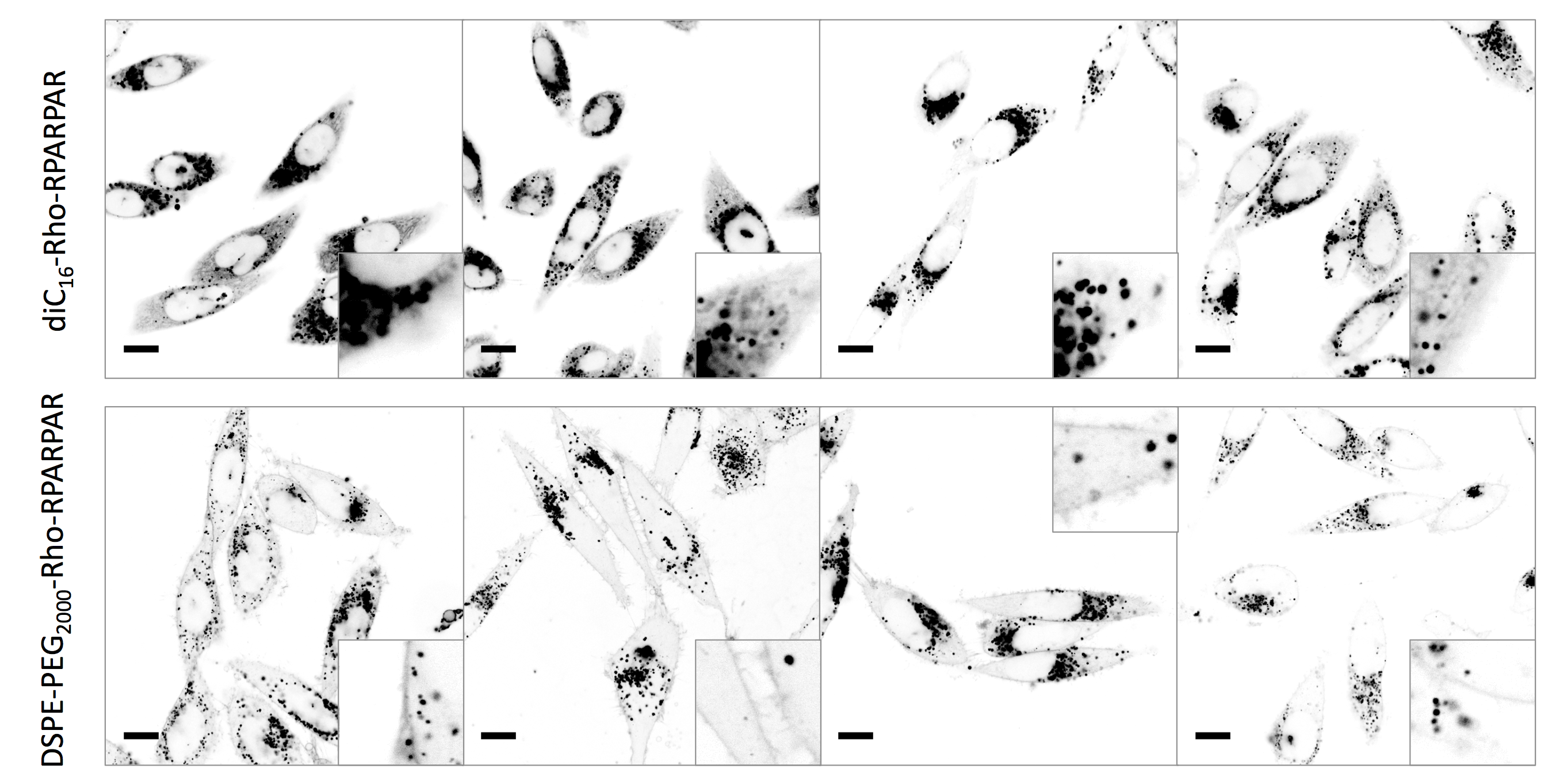

Supplement: Figure S7 — Membrane association after 24 h depends on PA architecture. PPC-1 cells pulsed 1 hour with diC16-Rho-RPARPAR (2) or DSPE-PEG2000-Rho-RPARPAR (4) and chased for 24 hours. A fraction of PA 4 was present on plasma membranes; in contrast, no PA 2 was detected on the plasma membrane. Confocal micrographs were processed to highlight membrane presence (or absence) of PAs. (TIFF) [file pone.0054611.s007.tiff]
